# 'Libyan Trial': a verdict running counter to scientific evidence

**DOI:** 10.1186/1742-4690-3-98

**Published:** 2006-12-27

**Authors:** Eric Fleutelot

**Affiliations:** 1 Head of International Programs, Sidaction, Paris, France

## Abstract

Sidaction's appeal regarding the sentencing of medical personnels in the Libyan-HIV infection cases.

## Commentary

A court in Tripoli, Libya, sentenced five Bulgarian nurses and a Palestinian doctor to death for intentionally infecting 426 Libyan children with HIV at Al Fateh Children's Hospital in Benghazi, Libya.

The six medical workers, along with the children and the families concerned, were involved in a political trial and are victims of a severe infringement of the most elementary human rights. Although new genetic evidence published earlier this month in the journal Nature [1] found that the HIV outbreak at the hospital began up to three years before the medical workers arrived at the facility, the Libyan government has decided to deny scientific proof and carry on a bloody strategy to fight against AIDS.

Pr. Hakima Himmich, member of Sidaction's Board and President of Moroccan Ngo ALCS, said that "it is an appalling story! These people are definitely victims of a political manipulation." Mr. Pierre Bergé, President of Sidaction, also declared "we're dealing here with a new example of collective cowardice". Sidaction denounces this trial as a parody of justice, which cannot hide the total incapacity of the Libyan Government to promote an appropriate politics of prevention and take care of people who are ill secondary to HIV infection-adults and children-in an appropriate way. It is useful to recall that the majority of Libyan people living with AIDS who are in need of an antiretroviral treatment can still not afford it.

Sidaction would like to stress that the fight against AIDS can only be carried on by respecting human rights, which includes the access to prevention, to appropriate care and to antiretroviral treatments. Sidaction appeals to the Libyan authorities to immediately release the six medical workers and commit to the fight against AIDS in an effective manner, by respecting human rights, and in particular the rights of people living with AIDS.
